# Prospective Use of Brown Spider Venom Toxins as Therapeutic and Biotechnological Inputs

**DOI:** 10.3389/fmolb.2021.706704

**Published:** 2021-06-17

**Authors:** Luiza Helena Gremski, Fernando Hitomi Matsubara, Nayanne Louise Costacurta Polli, Bruno Cesar Antunes, Pedro Henrique de Caires Schluga, Hanna Câmara da Justa, João Carlos Minozzo, Ana Carolina Martins Wille, Andrea Senff-Ribeiro, Silvio Sanches Veiga

**Affiliations:** ^1^ Department of Cell Biology, Federal University of Paraná, Curitiba, Brazil; ^2^ Production and Research Center of Immunobiological Products, State Department of Health, Piraquara, Brazil; ^3^ Department of Structural, Molecular Biology and Genetics, State University of Ponta Grossa, Ponta Grossa, Brazil

**Keywords:** *Loxosceles*, proteins, peptides, recombinant proteins, venom

## Abstract

Brown spider (genus *Loxosceles*) venoms are mainly composed of protein toxins used for predation and defense. Bites of these spiders most commonly produce a local dermonecrotic lesion with gravitational spread, edema and hemorrhage, which together are defined as cutaneous loxoscelism. Systemic loxoscelism, such as hematological abnormalities and renal injury, are less frequent but more lethal. Some *Loxosceles* venom toxins have already been isolated and extensively studied, such as phospholipases D (PLDs), which have been recombinantly expressed and were proven to reproduce toxic activities associated to the whole venom. PLDs have a notable potential to be engineered and converted in non-toxic antigens to produce a new generation of antivenoms or vaccines. PLDs also can serve as tools to discover inhibitors to be used as therapeutic agents. Other *Loxosceles* toxins have been identified and functionally characterized, such as hyaluronidases, allergen factor, serpin, TCTP and knottins (ICK peptides). All these toxins were produced as recombinant molecules and are biologically active molecules that can be used as tools for the potential development of chemical candidates to tackle many medical and biological threats, acting, for instance, as antitumoral, insecticides, analgesic, antigens for allergy tests and biochemical reagents for cell studies. In addition, these recombinant toxins may be useful to develop a rational therapy for loxoscelism. This review summarizes the main candidates for the development of drugs and biotechnological inputs that have been described in Brown spider venoms.

## Introduction

Brown spiders are well-adapted arthropods with more than 150 species distributed in all continents (World Spider Catalog. Version 22.0, 2021). In many places, the accidents with these venomous spiders are considered a public health issue, such as in some regions of South America (Da Silva et al., 2004). Few countries have a compulsory notification system that collect the number of cases of loxoscelism, which is why the number of cases around the world is probably underestimated (Lopes et al., 2020). Even in Brazil, which has a suitable notification system, this amount is possibly underrated mainly because the causative specimen is usually not identified. Nevertheless, almost 8,800 accidents with Brown spiders were reported in Brazil in 2019 and more than 80% of these cases occurred in the Southern region of Brazil. Between 2010 and 2019, almost 80,000 accidents were reported in Brazil (Ministério da Saúde, 2020). In Mexico, envenomation caused by *Loxosceles* spider bites is considered a public health problem as about 3,000 accidents with these spiders are annually reported (Sanchez-Olivas et al., 2011). These spiders are not aggressive and their bites usually occur when these animals feel threatened after being compressed against the skin (Futrell, 1992). *Loxosceles* spiders are small animals that produce minute amounts of venom. They use their venom primarily to kill or paralyze their prey—usually insects—and to deter possible predators, such as human beings (De Castro et al., 2004; Hogan et al., 2004; Chaim et al., 2011). The toxins that comprise this potent venom are highly active against mammalian tissues and trigger local and systemic signs that can vary widely. Victims usually develop a cutaneous lesion near the bite site with initial edema, erythema and itching followed by dermonecrosis with a gravitational spread, which is the hallmark of loxoscelism. The emergence of a systemic commitment is less frequent but is the main cause of deaths. Acute renal failure and hematological disturbances, such as intravascular hemolysis, figure as the major systemic complications of loxoscelism (Da Silva et al., 2004).

Loxoscelism is related to the biological activity of a range of toxins present in the brown spider venoms. Most of these toxins are peptides and proteins mostly exhibiting molecular masses between 5–40 kDa (Veiga et al., 2000; Gremski et al., 2010). One family of enzymes, the phospholipases D, is responsible for most local and systemic effects seen after envenoming. They cleave various types of phospholipids and generate biologically active lipids, which, after all, cause an exacerbated inflammatory response and tissue damage (Gremski et al., 2020). Astacin-like metalloproteases and hyaluronidases are also present. They target extracellular matrix (ECM) components such as fibronectin, entactin, proteoglycans and hyaluronan. Hyaluronidases are involved in the noxious activity of Brown spider venoms by acting as spreading factors of other toxins, enhancing, for example, the gravitational spread of dermonecrotic lesion (Ferrer et al., 2013). A Translationally Controlled Tumor Protein (TCTP) is also part of the venom, and acts as a histamine-releasing factor. Together with an allergen from the cysteine-rich secretory protein (CRISP) family, TCTP contributes to the allergic and inflammatory response of cutaneous loxoscelism (Sade et al., 2012; Boia-ferreira et al., 2019; Justa et al., 2020). A serine protease inhibitor from the Serpin superfamily was also described in a *Loxosceles* venom and, although its role as a toxin remains to be elucidated, the biological activities of this inhibitor indicate a great potential for drug development (Graeff et al., 2019). A great amount of knottins is also reported and, although their role in loxoscelism remains unknown, their insecticidal activity was already proved, which makes these molecules promising candidates as agents for the control of insect pests (Matsubara et al., 2017).

General data acquired from venom gland transcriptomes and venom proteomes elucidated the toxins’ expression profile of many *Loxosceles* species (Machado et al., 2005; Fernandes-Pedrosa et al., 2008; Dos Santos et al., 2009; Gremski et al., 2010; Trevisan-Silva et al., 2017; Calabria et al., 2019). In addition, these screening efforts allowed the description of coding sequences of less expressed toxins, which enabled the production of these molecules as recombinant proteins (Sade et al., 2012; Ferrer et al., 2013; Graeff et al., 2019; Justa et al., 2020; De-Bona et al., 2021). In fact, some toxins, such as wild-type and mutated/inactive phospholipases D and astacin-like metalloproteases were recombinantly expressed before the first screening reports (Fernandes Pedrosa et al., 2002; Kalapothakis et al., 2002; Chaim et al., 2006; Da Silveira et al., 2007; Kusma et al., 2008). However, the production of other recombinant proteins, i.e., hyaluronidases, TCTP, ICK peptides, allergen and serpin, was only possible after the initial descriptions of their coding sequences (Sade et al., 2012; Ferrer et al., 2013; Matsubara et al., 2017; Graeff et al., 2019; Justa et al., 2020). Recombinant chimeric proteins consisting of *Loxosceles* toxins’ epitopes were also produced (Mendes et al., 2013; Lima et al., 2018; Souza et al., 2018; Calabria et al., 2019). The expression model differs for each recombinant *Loxosceles* toxin since some of them are produced as soluble and active proteins by prokaryotic expression systems, while others require a more complex model to achieve the desired production protocol (Boia-ferreira et al., 2019; Graeff et al., 2019; Gremski et al., 2020; Justa et al., 2020; De-Bona et al., 2021). As the purification of toxins from Brown spider venoms usually yields tiny amounts of proteins, the production of recombinant toxins allowed their further characterization. Due to this massive characterization of *Loxosceles* venom components and to the possibility of producing them as recombinant proteins, these molecules have proven to be valuable tools for their application as antigens to produce improved antivenoms and a vaccine, and as useful scaffolds for the development of specific inhibitors, drugs and biological agents.

## A Rational Therapy for Loxoscelism Based on Venom Proteins

Although accidents with Brown spiders are frequent and occasionally severe, a definitive treatment for loxoscelism is not well established. As depicted in Table 1, a few therapies are available to manage patients with loxoscelism. As shown, *Loxosceles* antivenom is the only specific treatment against loxoscelism and its use is the standard recommendation in several countries (Lévano and Fernández, 2004; Ministerio de Salud, 2012; Bermúdez-Méndez et al., 2018; Ministério da Saúde, 2020). However, experimental studies with commercial products are limited to animals, and demonstrate that antivenom reduces cutaneous lesions in rabbits and protects mice of lethality induced by venom (Gomez et al., 1999; Pauli et al., 2009; Fingermann et al., 2020). Currently, clinical studies with available antivenoms are scarce (Fingermann et al., 2020) and some difficulties to produce them are reported. Spider capture and venom extraction, for instance, are laborious and expensive procedures that restrict the antivenom production. In addition, animal suffering is a common inconvenience in the immunization process. Considering this, the search for novel therapies and new methods of antivenom production are required and, fortunately, technological innovation is a reality that will certainly contribute to this improvement.

**TABLE 1:** Available therapies used to treat loxoscelism—summarization of their features and basic references on the matter.

Available therapies	Purpose	Target	Limitations	References
Anti-inflammatory drugs—mainly dapsone (topic and oral)	Reduce the massive inflammatory reaction mainly triggered by venom PLDs and refrain the development of dermonecrosis	Cutaneous lesion	Non-specific	(Manríquez and Silva. (2009), Miranda et al. (2021))
			Adverse effects of dapsone, such as anemia, colostasic jaundice, hepatitis, leukopenia, which can be difficult to differentiate as drug effect versus potential visceral compromise of the bite	
Hyperbaric oxygen therapy	Treat nonhealing wounds caused by *Loxosceles* venom	Cutaneous lesion	Non-specific	(Beilman et al. (1994), Hobbs et al. (1996))
			Aims only patients with nonhealing wounds	
			High-cost method, not often available	
Antibiotics	Manage possible secondary infection	Cutaneous lesion	Non-specific	(Miranda et al. (2021))
			Does not prevent the development of the normal lesion induced by the venom	
Therapeutic plasma exchange	Removes molecular components from the blood, possibly molecules related to the complement system	Systemic injury	Non-specific	(Said et al. (2014); Abraham et al. (2015))
			Targeted to specific patients, such as those presenting hemolysis	
			Needs further investigation	
			High-cost method	
Antiloxoscelic serum produced with venoms of *Loxosceles gaucho*, *L. intermedia* and *L. laeta* and Antiarachnidic serum produced with venoms of *L. gaucho*, *Phoneutria nigriventer* and *Tityus serrulatus*	Neutralize circulating venom toxins, reducing their action upon target tissues, such as kidneys, blood and liver	Systemic injury (main) and Cutaneous lesion	Efficacy depends on early application (up to 6 h after the bite) High-cost method Animal welfare issues along the production process Present side effects that vary from rashes to severe adverse reactions (anaphylaxis, anaphylactoid reactions, serum sickness and death)	(Barbaro et al. (1996), De Almeida et al. (2008), Pauli et al. (2009))

### Antivenom and Monoclonal Antibodies for the Treatment of Loxoscelism

The use of serum therapy to manage patients bitten by venomous animals dates from the last decades of the 19th century (Calmette, 1986; Hawgood, 1992; Pucca et al., 2019). Despite the advancements, current *Loxosceles* antivenoms are still based on plasma-derived fragments of immunoglobulins from animals hyper-immunized with venom. Since the purification of large amounts of venom is one of the major hitches in this process, the first efforts to use recombinant toxins as immunogens were made. As phospholipases D (PLDs) can trigger most of the toxic effects caused by crude venom, the first studies focused on these toxins to replace the venom in the antivenom production (Chaim et al., 2006; Kusma et al., 2008; Chaves-Moreira et al., 2009, 2011; Gremski et al., 2014, 2020; da Silva et al., 2021).

The PLD/smase I from *L. laeta* was the first recombinant toxin used as an antigen in an immunization protocol using rabbits. The antiserum raised against this toxin blocked the development of dermonecrosis induced by *L. laeta* venom in rabbits (Fernandes Pedrosa et al., 2002). In addition, a mixture of recombinant PLDs of two North American species *L. reclusa* (Lr1N), and *L. boneti* (Lb1C), and one South American species *L. laeta* (L11N; L12C) was later tested (Olvera et al., 2006). Comparative analyses between the commercial Peruvian antivenom, produced by hyperimmunization of horses with *L. laeta* (Peru) venom, and a serum produced by hyperimmunization of horses with a PLD of *L. intermedia* (Brazil) (rLiD1) demonstrated a limited protection when only the recombinant toxin was used as antigen. The anti-venom performed 100% of protection against the lethality in mice induced by *L. laeta* and *L. intermedia* venoms. Nevertheless, while anti-PLD serum fully protected the animals from *L. intermedia* venom, a partial protection (75%) was observed against *L. laeta* venom (Duarte et al., 2015). Both sera completely neutralized the dermonecrotic and hemorrhagic activities, but partially inhibited the edematogenic activity of *L. laeta* venom in rabbits (Duarte et al., 2015).

In 2008, the production and purification of a polyvalent serum produced in horses with PLDs from *L. intermedia* (rP1 and rP2) and from *L. laeta* (smase I) were patented (patent 0404765-6 INPI) (De Almeida et al., 2008). The efficacy of this serum in reducing dermonecrotic lesions was compared to the commercial anti-arachnid serum (produced in horses with a mixture of venoms from *Loxosceles gaucho*, *Phoneutria nigriventer* and *Tityus serrulatus*). The anti-PLD serum showed to be more active than anti-arachnidic serum against dermonecrosis and hemolysis caused by *L. intermedia* and *L. laeta* venoms and had the same protection against the venom of *L. gaucho* (De Almeida et al., 2008). A recombinant PLD of *L. gaucho* (LgRec1) also demonstrated to be highly immunogenic, and the previous incubation of the sera from rabbits immunized with this protein fully inhibited dermonecrosis triggered by *L. gaucho* venom (Magalhães et al., 2013). These studies showed that PLDs are good candidates to replace crude venoms for antivenom production. However, it is important to highlight that *Loxosceles* venoms are composed by diverse homologous isoforms of PLDs and other molecules that may play an important role in the cutaneous and systemic loxoscelism. In addition, the cross-neutralization is not observed among PLDs from all *Loxosceles* species. Finally, active PLDs are toxic to the injected animals and, as with the venom, continuous immunizations with these proteins can greatly affect the health of immunized animals and reduce their lifespan.

In this regard, efforts have been made to improve the protocol of immunization. The use of synthetic peptides with amino acid sequences corresponding to epitopes or antigenic regions of toxins could also be applied to antivenom preparation. For instance, antigenic regions of a PLD of *L. intermedia* (LiD1) were synthesized and used to immunize rabbits. The purified IgG pre-incubated with LiD1 reduced dermonecrotic, hemorrhagic and edema activities of this protein by 82, 35 and 35% respectively, but the co-immunization of these peptides with LiD1 improved the neutralization capacity of IgGs against LiD1. However, the protection against the venom was not evaluated (Felicori et al., 2009). One chimeric protein (Lil) containing epitopes of *L. intermedia*, *L. laeta* and *L. gaucho* PLDs was also tested as an immunogen. Around 40% of necrosis, hemorrhage and edema were neutralized when *L. intermedia* venom was preincubated with anti-Lil serum (Souza et al., 2018). Better results were found when a recombinant chimeric protein (rCpLi) formed by a fusion of three epitopes of LiD1 was used: the previous incubation of anti-rCpLi IgG with rLiD1 partially neutralized its dermonecrotic (95%), hemorrhagic (75%) and edematogenic (10%) effects (Mendes et al., 2013). An immunization schedule in horses that combined rCpLi and *Loxosceles* venoms as immunogens produced sera that fulfilled the efficacy tests (100% of reduction in dermonecrosis elicited by *L. intermedia* venom in rabbits). The protocol included three initial injections of a mixture of *L. intermedia*, *L. laeta* and *L. gaucho* venoms, six additional injections with rCpLi and a re-immunization schedule consisting of three boosters of the venom mixture. The sera of horses that received only rCpLi on immunizations performed around 50% of reduction in dermonecrosis (Figueiredo et al., 2014). As mentioned earlier, loxoscelic venoms have other proteins that play a role in loxoscelism, i.e., metalloproteinases, serine proteases, hyaluronidases, TCTP and allergens (Feitosa et al., 1998; Veiga et al., 2000; Da Silveira et al., 2007; Trevisan-Silva et al., 2010; Ferrer et al., 2013; Justa et al., 2020; De-Bona et al., 2021). Thus, the inclusion of these toxins in the production of sera turns out to be a strategy to enhance the efficacy of antivenoms. Therefore, recombinant chimeric proteins containing selected epitopes of some of these proteins were created. For instance, epitopes from a smase I of *L. laeta*, a metalloprotease (LALP-1) and a hyaluronidase (LiHyal) of *L. intermedia* were fused with rCpLi forming a multiepitope chimeric protein (rMEPLox) which was used as an immunogen in rabbits. Previous incubation of anti-rMEPLox sera protected 60% of mice from lethal effects of *L. intermedia* venom (Lima et al., 2018). In addition, pre-incubation of this venom with anti-rMEPLox IgGs reduced 60% of the hyaluronidase activity of venom (Lima et al., 2018). Another chimeric protein (LgRec1ALP1) which included antigenic regions of a PLD (LgRec1) and a metalloprotease (LgALP1) of *L. gaucho* venom was also evaluated as antigen (Calabria et al., 2019). Previous incubation of anti-LgRec1ALP IgGs with the venoms of *L. gaucho*, *L. intermedia*, *L. laeta* reduced the dermonecrotic effects of these venoms in 100, 79 and 68%, respectively (Calabria et al., 2019). *In vitro*, those IgGs reduced the platelet aggregation and the proteolytic action of metalloproteases of the three venoms (Calabria et al., 2019).

**FIGURE 1:**
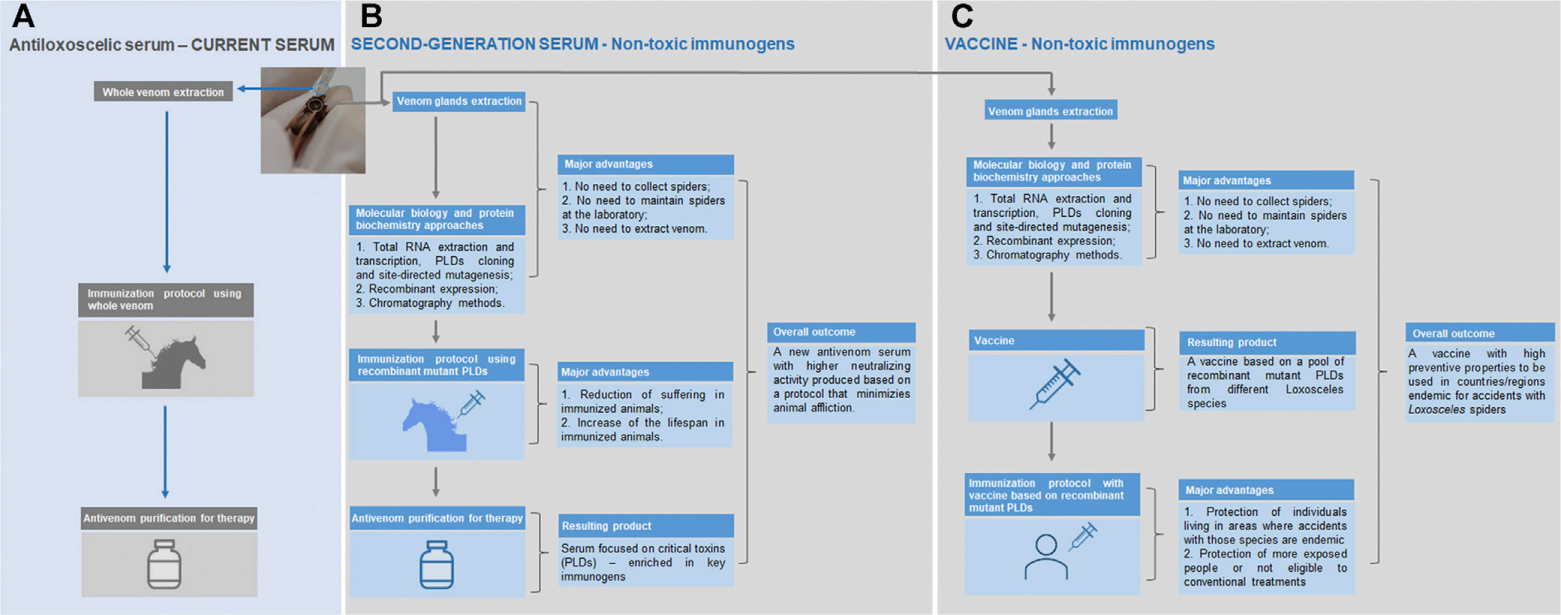
Current antivenom therapy (antiloxoscelic serum) and the new approaches using non-toxic immunogens (second-generation serum and vaccine). **(A)** The current serum is produced using the whole venom extracted by a qualified staff from spiders captured in nature; the immunized animals to produce the serum develop unwanted reactions derived from the whole venom toxicity (pain and swelling at the inoculation site and, in some cases, suppuration; inappetence and episodes of mild and transient fever may appear); although the current protocol is well established, it is subjected to a certain irreproducibility, since the yield of venom extractions can vary and this could have a negative impact on the periodic animals’ immunization procedures. **(B)** The strategy regarding the second-generation serum combines cost saving (no expenses related to spider collection missions and maintenance of these animals in captivity), staff safety (no recurrent manipulation of spiders) and easy management of the production process (it does not depend on the efficiency of the whole venom extraction); most importantly, the method for the generation of the new antivenom assures the depletion of adverse signs and symptoms in the immunized animals (mutant PLDs used are not tissue-destructive as the native PLDs) and the serum efficacy is expected to be higher, since it is produced using the molecules responsible for most of the noxious effects seen in the loxoscelism—the PLDs. **(C)** The development of a vaccine based in recombinant mutant PLDs from different *Loxosceles* species will be important for the protection of individuals living in areas where accidents with those species are endemic, as well as in people more exposed or not eligible to conventional treatments.

Despite the use of chimeric proteins and linear epitopes with antigenic regions in immunization protocols has demonstrated promising results, structural analyses of epitope-antibody interactions have shown that over 90% of epitopes in proteins are conformational and do not react with any peptide fragment derived from the parent protein (Van Regenmortel, 1996). In this sense, the recombinant expression of PLDs, which is well-established, allowed the production of non-toxic molecules that may nonetheless elicit protective humoral responses while minimizing envenomation and suffering of animals used in antivenom production. The fusion of a β-galactosidase tag with a PLD (Li-rec) eliminated its toxicity; the application of this protein as an immunogen produced a serum that neutralized 2.5 LD50 of *L. intermedia* venom per ml of serum in a murine lethality model (Kalapothakis et al., 2002; Araujo et al., 2003). Comparative analyses between the protection of animals immunized with recombinant active (rLlPLD1) or inactive (rLlPLD2) isoforms of *L. laeta* PLDs showed that both protocols reduced the development of dermonecrotic lesions induced by *L. laeta* venom at similar levels, thus reinforcing that the antigenic potential of these enzymes is not related to their activity (Catalán et al., 2011). Molecular engineering enabled the production of mutated PLDs from *L. intermedia*, *L. laeta* and *L. gaucho* venoms with appropriate conformational structure, but devoid of enzymatic activity, opening the possibility to apply these proteins as immunogens to the development of antivenoms or vaccines (Vuitika et al., 2016; da Silva et al., 2021). Figure 1 illustrate this potential of such toxins. Moreover, a hyaluronidase (LiHyal2) and an allergen (LALLT) from *L. intermedia* venom were recombinantly expressed and may be used to enhance the range of antigens for antivenom production (Justa et al., 2020; De-Bona et al., 2021).

Another strategy that has been explored is the production of monoclonal antibodies (mAbs) that cross-react with PLDs and neutralize toxic effects of Brown spider venoms. One of these mAbs, LimAb7, was initially produced by immunization with the venom of *L. intermedia* and efficiently neutralized the dermonecrotic activity of *L. intermedia* venom (Alvarenga et al., 2003). However, this mAb did not cross-react either with the venoms of *L. gaucho* or with the venoms of the Peruvian and Brazilian *L. laeta* (Alvarenga et al., 2003). LimAb7 was then re-engineered to a recombinant diabody, which was efficient in neutralizing the sphingomyelinase and hemolytic activities of *L. intermedia* venom, although it exhibited a limited stability in this dimeric configuration (Karim-Silva et al., 2016). Recently, humanized recombinant single-chain antibody fragments (scFvs) based on LimAb7 were produced and shown to inhibit the hemolytic effect of *L. intermedia* venom in the presence (68% of inhibition) or absence (>90%) of the complement system (Karim-Silva et al., 2020).

Aiming to overcome the limited cross-reaction of LimAb7 with different *Loxosceles* species, LiD1mAb16 was produced by immunization of mice with the PLD LiD1 (Dias-Lopes et al., 2014). In fact, this novel mAb recognized at least 25 proteins in each tested venom, i.e., *L. intermedia*, *L. laeta* and *L. gaucho* venoms, and interacts with an epitope inside the catalytic loop of LiD1. Pre-incubation of LiD1mAb16 with LiD1 protected the rabbits against dermonecrosis and local hemorrhage induced by the toxin with 80% of neutralization, while protection against edema achieved 48% of neutralization (Dias-Lopes et al., 2014). The chimeric protein rMEPlox mentioned earlier was also used as antigen to produce a monoclonal antibody named Lox-mAb3. This mAb recognized a metalloprotease of *L. intermedia*, cross-reacted with metalloproteases of *L. laeta* and *L. gaucho,* and neutralized the fibrinogenolytic activity of *L. intermedia* venom, which may decrease hemorrhagic disturbances caused by *Loxosceles* envenomation (Costa et al., 2020).

Thus, as addressed, different strategies have been explored by toxinologists to acquire rationally designed tools to improve the management of patients bitten by brown spiders. The development of novel antivenoms based on recombinant toxins, chimeras or synthetic peptides are all promising approaches. Monoclonal antibodies and their derivatives are also tools with potential application to treat patients affected by loxoscelism.

### Vaccines to Prevent Loxoscelism

Alternative strategies, such as vaccination protocols, have been studied to prevent loxoscelism. In this sense, various non-toxic immunogens based on *Loxosceles* PLDs were tested. For instance, some synthetic peptides similar to epitopes or antigenic regions of toxins were tested as immunogens in protection experiments. Linear and conformational epitopes from PLDs and chimeric proteins containing epitopes of different toxins were evaluated. Initially, rabbits immunized with a pool of non-toxic peptides corresponding to antigenic regions of LiD1 that produced a partially neutralizing serum (described in 2.1) was also tested for protection. When these animals were challenged with recombinant LiD1 (LiD1r), a protection of 40–50% was observed against dermonecrotic and hemorrhagic activities of this toxin, and 10% of protection was reached against the edematogenic effect (Felicori et al., 2009). In addition, a continuous B-cell epitope (27-mer peptide) corresponding to a region of LiD1 involved in the active site were synthesized and used as immunogen in mice and rabbits (Dias-Lopes et al., 2010). Immunized mice challenged with 1.5 LD50 of LiD1r presented 75% of protection against the lethal activity of this toxin. This immunization protocol in rabbits elicited about 70% of protection against dermonecrotic and hemorrhagic activities of LiD1r (1 MND/kg), and a low protection against edema (Dias-Lopes et al., 2010). Based on the findings regarding the epitope of LiD1 that is recognized by the monoclonal antibody LimAb7, two mimotopes were synthesized, entrapped into liposomes and used as immunogens in rabbits. These animals were challenged with *L. intermedia* venom, and immunization protocol showed 60% of protection against dermonecrosis, 80% against hemorrhage and 30% against edema (Moura et al., 2011). The aforementioned chimeric protein rMEPLox, containing epitopes from PLDs of *L. laeta* and *L. intermedia*, and from a metalloprotease and a hyaluronidase from *L. intermedia*, was also evaluated for its vaccinal potential. rMEPLox induced an immune response that completely blocked the dermonecrosis induced by 3.35 MND of *L. intermedia* venom (Lima et al., 2018).

Non-toxic PLDs with a three-dimensional structure similar to active isoforms were also tested as immunogens, following the notion that most epitopes in proteins are discontinuous and do not react peptide fragments from the parent protein (Van Regenmortel, 1996). For example, the aforementioned non-toxic PLD fused with β-galactosidase (Li-rec) were used in a vaccination protocol in mice, and fully protected the animals against the lethal effects of 2.5 LD50 of *L. intermedia* venom. The same study also showed that rabbits immunized with Li-rec were partially protected against dermonecrosis 120 days after the last immunization dose, indicating that this protein generates long lasting antibodies (Araujo et al., 2003). The inactive PLD isoform rLIPLD2 from *L. laeta* were also evaluated for this purpose. The protection of rabbits against the dermonecrotic activity of *L. laeta* venom was evaluated in animals previously vaccinated with rLIPLD2, and a good protection was observed (Catalán et al., 2011). These studies support the use of non-toxic recombinant proteins in vaccination protocols against loxoscelism. In this sense, the mutated and inactive recombinant PLDs of *L. intermedia*, *L. laeta* and *L. gaucho* mentioned above could be used as antigens in a new-generation vaccination protocol in areas where accidents with those species are endemic, as well as in people more exposed or not eligible to conventional treatments, as depicted in Figure 1 (Vuitika et al., 2016; da Silva et al., 2021).

## Venom Peptides and Proteins as Potential Novel Drugs

Brown spider venoms are complex chemical cocktails with proteins and peptides with quite specific biochemical and biological activities. Such activities can be further explored to apply these toxins as therapeutic or biological agents optimized for numerous purposes (summarized in Table 2). In the next items, such potential applications will be explored, based on the known biological activities of these toxins and on the literature regarding these proteins.

**TABLE 2:** Summarization of the protein content of *Loxosceles* spiders’ venoms—classes of toxins and potential applications of these molecules to manage the loxoscelism and to develop drugs and biological/biotechnological inputs.

Brown spider venom molecules	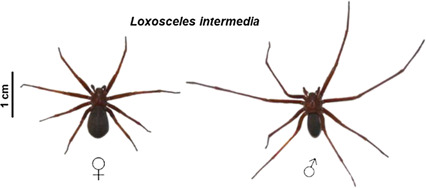 Potential applications and basic references on the matter
Phospholipases D (30–35 kDa)	• New therapies to treat loxoscelism
	1. Development of a second generation antivenom produced from mutant PLDs or engineered peptides/chimeric proteins based on PLDs (Mendes et al. (2013), Vuitika et al. (2016), Lima et al. (2018), Calabria et al. (2019), da Silva et al. (2021))
	2. Development of monoclonal antibodies that cross-react with venom PLDs (Alvarenga et al. (2003), Dias-Lopes et al. (2014), Karim-Silva et al. (2016), Costa et al. (2020))
	3. Development of a vaccine for areas where the accidents are endemic (Lima et al. (2018), da Silva et al. (2021))
	• Biotool to be used in studies regarding tumor cell biology (Wille et al. (2013), Siqueira et al. (2019))
	• Antimicrobial drug (Segura-Ramírez and Silva Júnior. (2018))
Serpins (44–46 kDa)	• Development of anticoagulant drugs (Schemczssen-Graeff et al. (2021))
	• Development of drugs for cancer treatment (Schemczssen-Graeff et al. (2021))
	• As potential antibacterial (Costa et al. (2014)) and insecticide (Clemente et al. (2019)) molecules
	• As tools for cell biology studies regarding proliferation, migration and control for protein half-life (Marathe et al. (2019))
Allergens (42–45 kDa)	• Development of skin and blood allergic sensitivity test (Linhart and Valenta. (2012))
	• Therapeutic input to be used in desensitizing protocols to treat allergic patients (Linhart and Valenta. (2012))
	• Biological inputs to be used in specific immunotherapy protocols (Linhart and Valenta. (2012))
Hyaluronidases (44–48 kDa)	• Drug diffusion enhancer (Weber et al. (2019))
	• Development of drugs/inputs for cancer treatment (Khan et al. (2018))
	• Development of inputs for aesthetic procedures (Weber et al. (2019))
Knottins (ICK peptides) (5–10 kDa)	• Development of effective bioinsecticides (Fitches et al. (2012), Bonning and Chougule. (2014), Herzig et al. (2016), Matsubara et al. (2017), King. (2019))
	• Development of analgesic drugs (Cardoso et al. (2017), Dongol et al. (2019))
	• Development of antifungal (Ayroza et al. (2012)), antiarrhythmic (Bode et al. (2001)) and antimalarial (Choi et al. (2004)) drugs
	• Biological inputs to be used as imaging agents for tumor detection (Moore et al. (2013); Kintzing and Cochran. (2016))
TCTP (22 kDa)	• Biotool to be used in studies regarding parasites biology or biological input to be used in the development of vaccines against parasites (Bhisutthibhan et al. (1998); Taylor et al. (2015))
	• Development of drug delivery systems (Bae et al. (2018))
	• Biotechnological input to be used as dental restorative material (Wanachottrakul et al. (2011), Sangsuwan et al. (2015), Kedjarune-Leggat et al. (2020))

### Serine Protease Inhibitors: Candidates in the Development of Biochemical Reagents, Insecticides, Antibacterial and Antitumoral Drugs

Serine protease inhibitors have been described in the literature as molecules with many functional activities regarding both physiological and pathological processes, not to mention the vast potential for biotechnological exploitation (Gettins, 2002; Huntington, 2011; Lucas et al., 2018). Although little studied, serine protease inhibitors in the venoms of *Loxosceles* spiders have been reported in different species, which shows the biological conservation and the importance of this components in the venoms. The first finding indicating the existence of serine protease inhibitors in *Loxosceles* venoms was reported in a study using the venom of *L. reclusa* (North America), in which the presence of a complement inhibitor was identified after venom fractionation using Sephadex G-75 exclusion chromatography (Kniker et al., 1969). Later, other more specific and directed studies identified these inhibitors in different species endemic in Brazil and South America. Omics methods have reported the presence of serine protease inhibitors in the venoms of *L. laeta* (Fernandes-Pedrosa et al., 2008), *L. intermedia* (Dos Santos et al., 2009; Gremski et al., 2010) and, more recently, in *L. gaucho* venom (Calabria et al., 2019), always as low expressed proteins. However, the presence of serine protease inhibitors in the venoms of different species of *Loxosceles* spiders is not sufficient to prove that these molecules are in fact toxins and have harmful activities with clinical correlation to the envenoming. In this context, the inhibitory activity of plasma coagulation induced by thrombin was associated with a recombinant serpin of *L. intermedia* (Schemczssen-Graeff et al., 2021), which could suggest that these venom molecules may contribute with the delay of plasmatic coagulation caused by *Loxosceles* venom (Zanetti et al., 2002) and, consequently, increasing the hemorrhagic disturbances during loxoscelism, such as local hemorrhage observed in the cutaneous lesion (Ospedal et al., 2002). As venom components, serine protease inhibitors may also act, for instance, as protectors, preventing the proteolysis of self-proteins/toxins and then increasing the half-life of them; this contributes to the integrity of the venom, keeping its potentiality in order to be used during hunting preys, defense against predators or in accidents with humans (Dos Santos et al., 2009; Gremski et al., 2010).

The great advance regarding studies of serine protease inhibitors found in *Loxosceles* venoms emerged with the cloning and recombinant expression of serine protease inhibitor from *L. intermedia* in *Spodoptera frugiperda* cells using baculovirus technology. This molecule was called LSPILT, from *Loxosceles* Serine Protease Inhibitor-Like Toxin (Schemczssen-Graeff et al., 2021). Authors have shown that this protein has a molecular mass of 46 kDa, and the amino acid sequence shows the presence of the signature for the Serpin super family members. LSPILT is homologous to other serpins found in spiders of other genus, as well as in other arthropods. In addition, the immunoassays suggested the conservation of this class of molecules in *Loxosceles gaucho, L. laeta* and *L. intermedia* venoms (Schemczssen-Graeff et al., 2021). The expression and purification of LSPILT yielded 8.0 mg/L of culture supernatant in its soluble form and without requiring refolding. LSPILT inhibits the activity of trypsin upon gelatin and vitronectin *in vitro*. LSPILT activity was also demonstrated by its inhibitory activity on the plasma coagulation induced by thrombin, inhibitory effect of lysis of *T. cruzi* trypomastigotes dependent on convertases (serino proteases) of complement system. Finally, LSPILT induced the inhibition of melanoma cell (B16-F10) proliferation and migration, events with great participation of serine proteases. These results all together has proven that LSPILT is a molecule with a broad spectrum of inhibitory activity on serine proteases (Schemczssen-Graeff et al., 2021).

These molecules are versatile and are efficient tools, which can be used to understand the participation of serine protease inhibitors in the context of envenoming or as endogenous proteolytic inhibitor of venom components. Also, LSPILT constitutes a tool to study structural aspects of serpin-family members through crystallography methods. LSPILT can be used as a prototype (taking into account the participation of serpins in a large number of biological events and in human health) for analyzes concerning biotechnological applicabilities, as previously pointed for other serpin family-members (Gaci et al., 2013). Regarding cell biology experimental studies, the recombinant LSPILT can be used as a prototype in protocols aiming to unveil how cells proliferate, migrate, undergo to apoptosis and control cellular protein half-life (Marathe et al., 2019). Regarding the control of agricultural infestations, LSPILT can be investigated as a potential input, since serpins have been shown as molecules able to inhibit digestive enzymes present in the gastrointestinal tract of insect, inducing an insecticide effect (Clemente et al., 2019). It has already been reported that serine protease inhibitors can exhibit antibacterial activities, which makes them potential antibacterial agents in the treatment of human diseases (Costa et al., 2014). Finally, LSPILT can be used as an useful reagent in different biochemical experimental protocols, such as serine protease ligands in affinity chromatography procedures, in the purification of proteases, or as an inhibitory agent during cell analysis or protein purification, avoiding undesirable proteolytic effects (Sabotič and Kos, 2012).

### Allergens: Potential Compounds to be Applied in the Diagnose and Treatment of Allergic Events

As mentioned above, the most common complications described in accidents caused by *Loxosceles* spiders are inflammatory reactions at the bite site, which can evolve to dermonecrosis between 24 and 48 h after accidents (Isbister and Fan, 2011; Malaque et al., 2011; Gremski et al., 2014; Chaves-Moreira et al., 2017). Signs of allergenic responses are also reported, and are characterized by swelling, itching, redness and cutaneous rash. These allergenic manifestations are not restricted to the bite site and usually emerge earlier when compared to inflammatory signs, and they clearly indicate the presence of allergenic toxins in the venoms (Makris et al., 2009; Isbister and Fan, 2011; Lane et al., 2011; Malaque et al., 2011; Justa et al., 2020). The presence of toxins theoretically responsible for allergic responses in the *Loxosceles* venoms was initially identified in the analysis of the transcriptome of *L. laeta* venom glands (Fernandes-Pedrosa et al., 2008). Subsequently, a proteomic study of the venom of *L. intermedia*, revealed the presence of a molecule with high identity to a protein similar to an allergen found in mites (Mite Allergen of Group 7) (Dos Santos et al., 2009), which was later confirmed by the analysis of *L. intermedia* venom gland transcriptome (Gremski et al., 2010). The study of the transcriptome of *L. gaucho* venom glands also reported transcripts related to allergens (Calabria et al., 2019).

The confirmation of toxins as allergens in the venoms of different *Loxosceles* species has finally been described by the cloning and recombinant expression of a toxin from *L. intermedia* (Justa et al., 2020)*.* The authors showed the presence of an allergen belonging to the CAP superfamily (Cysteine-Rich Secretory Proteins) with 42 kDa. This toxin was named LALLT (*Loxosceles* Allergen-Like Toxin), and it was produced by using a eukaryotic heterologous expression system (Sf9 insect cells combined to the baculovirus technology) (Justa et al., 2020). Research on amino acid alignments with other allergens, immunological reactivity using antibodies raised originally to LALLT or crude venoms from different *Loxosceles* species, and searches for high identity mRNAs sequences in other *Loxosceles* species showed that LALLT has biological conservation (Justa et al., 2020). Recombinant LALLT was able to trigger mast cells degranulation, in addition to cause leukocyte infiltration into the dermis of rabbits, induce paw edema and increase the vascular permeability in mice skin (Justa et al., 2020). Together, the experimental data acquired state this toxin as an allergic agent with clinical potential, which should be evaluated as a target in the treatment of loxoscelism, especially in injured patients with a history of previous allergies to arthropods.

As protein content in *Loxosceles* venoms is quite reduced (Sams et al., 2001; Binford and Wells, 2003; Da Silva et al., 2004; Senff-Ribeiro et al., 2008; Gremski et al., 2014), the production of recombinant LALLT in a eukaryotic model is a breakthrough regarding the characterization of toxins from *Loxosceles* spiders. It was the first recombinant *Loxosceles* toxin to be successfully produced in the mentioned model. The availability of correct-folded and functional isoforms of LALLTs will allow, in a near future, the crystallization of this toxin, providing data related to the structure/function relationship and the action mechanism of this molecule. In addition, with a functional eukaryotic recombinant model, we can exhaust the analysis of biochemical, biological, immunological and pharmaceutical activities for members of this family. Questions regarding the real allergenic potential of these toxins, other toxic activities that have not yet been described and cell receptors involved with the toxic activities can be answered using recombinant allergens produced according to the mentioned methodology.

The availability of the recombinant LALLT may enable its use as antigen for skin allergic sensitivity test, allowing the identification of sensitive populations and more rational treatments, reducing adverse reactions due to envenoming. They may also be used as antigens for blood allergic tests, seeking for reactivity with immunoglobulins present in the blood of patients and confirming a predisposition to allergies. In severe cases, recombinant LALLT may also be used in desensitizing protocols to treat allergic patients. Finally, recombinant LALLT can be used in specific immunotherapy protocols for related antigens, giving rise to protective vaccines against severe allergic attacks, as previously discussed for other allergenic factors (Linhart and Valenta, 2012).

### Hyaluronidases: Adjuvants to Enhance the Diffusion of Other Drugs and Agents to Correct Esthetic Filling Procedures

Together with mammalian hyaluronidases, venom hyaluronidases belong to the EC 3.2.1.35 group of enzymes and act as endo-β-*N*-acetyl-d-hexosaminidases. Their main substrate is hyaluronic acid (HA), but can also cleave chondroitin sulfate (Bordon et al., 2015). HA is abundant in the extracellular matrix (ECM) of mammal soft connective tissues (Girish and Kemparaju, 2007). Besides its structural basic functions in ECM, HA has been linked to more specific molecular functions, such as binding to ECM proteins and to particular cell receptors that mediate important physiological processes (Girish and Kemparaju, 2007).

Degradation of HA by hyaluronidase increases connective tissue permeability and decreases viscosity of body fluids. These enzymes are directly involved in the spread of venoms and toxins, and in important processes such as fertilization and cancer progression. Depolymerization of HA also impairs the ECM activity as a reservoir of growth factors (Cramer et al., 1994; Menzel and Farr, 1998; Kemparaju and Girish, 2006; Chao et al., 2007). Hyaluronidases have been described in several animal venoms like snakes, bees, scorpions, spiders, lizards and stingrays (Kemparaju and Girish, 2006; Magalhães et al., 2008; Ferrer et al., 2013). Hyaluronidases from venomous animals are commonly described as spreading factors once HA degradation increases the ECM permeability, rendering tissues highly permeable to the toxic components of venom (Girish and Kemparaju, 2007).

Multiple applications regarding mammalian hyaluronidases have already been described, one of which is related to the combined use with local anesthetics to enhance the diffusion of injected therapeutic drugs. On this subject, the application of a recombinant human hyaluronidase (rHuPH20) has recently gained prominence for the subcutaneous application of insulin, morphine, immunoglobulins and other pharmaceuticals (Weber et al., 2019). In different studies, it has been demonstrated that the administration of hyaluronidases with anti-cancerous drugs reduces the interstitial fluid pressure within the tumorous tissue and, as a spreading factor, these enzymes can also be used to enhance the penetration of oncolytic agents, potentiating the effectiveness of the therapy. The additive use of the hyaluronidases is not limited to the chemotherapy as they may also be used in combination with radioimmunotherapy (Khan et al., 2018). Furthermore, studies have shown the effectiveness of hyaluronidase as an adjuvant in neuroplastic procedures by reducing pain rating compared to other techniques (Helm and Racz, 2019). The injection of hyaluronidase is also used to correct unaesthetic overcorrections and to reverse chronic edema or vascular occlusion after HA-filler applications (Weber et al., 2019).

Nowadays, commercial formulations of exogenous hyaluronidases include purified forms from bovine (BTH) and ovine testicles (OTH) and recombinant human hyaluronidase (rHuPH20) (Searle et al., 2020). Animal derived hyaluronidases are extracted from testis tissue, and are generally purified by series of multiple precipitation, fractionation and filtration steps (Farooqui and Srivastava, 1979; Lyon and Phelps, 1981; Kaya et al., 2015). One of the limitations of these methods is that these extracts are often contaminated with proteases, immunoglobulin, and other elements, which can increase capillary permeability or IgE-mediated hypersensitivity reactions (Silverstein et al., 2012). The recombinant form of hyaluronidase is clinically available as Hylenex™ (Baxter, Deerfield, IL). The recombinant human hyaluronidase (rHuPH20) is a highly purified protein. The production uses Chinese hamster ovary cells (CHO) to express the human recombinant genetic engineered enzyme. This hyaluronidase is purified through a series of column chromatographies and other processing steps, generating a protein with less impurities and potential pathogens, reducing the risk of hypersensitivity reactions (Frost, 2007; Silverstein et al., 2012).

Venom hyaluronidases have been studied as purified enzymes (Poh et al., 1992; Girish et al., 2004; Morey et al., 2006; Horta et al., 2014) or as recombinant proteins mainly expressed in insect cells (Soldatova et al., 2007; Clement et al., 2012; De-Bona et al., 2021). In *Loxosceles* spider venoms, hyaluronidase activity was identified in six *Loxosceles* species (*L. rufescens, L. deserta*, *L. gaucho*, *L. intermedia*, *L. laeta* and *L. reclusa*) and some of these results were confirmed by subsequent proteome and transcriptome studies that described hyaluronidases or coding sequences for such enzymes in various *Loxosceles* venoms (Young and Pincus, 2001; Barbaro et al., 2005; Fernandes-Pedrosa et al., 2008; Dos Santos et al., 2009; Gremski et al., 2010; Trevisan-Silva et al., 2017; Siqueira et al., 2019). Initially, a recombinant hyaluronidase from *L. intermedia* venom was produced in a prokaryotic model, purified, refolded and characterized. LiHyal, as it was named, was able to degrade hyaluronic acid and chondroitin sulfate *in vitro*, and biological assays revealed that this recombinant toxin was able to increase erythema, ecchymosis and dermonecrotic effects when associated with a recombinant phospholipase D, indicating that this protein acts as spreading factor (Ferrer et al., 2013). Later, a similar isoform was expressed in baculovirus-infected insect cells as a soluble and active recombinant hyaluronidase, named LiHyal2, with similar *in vitro* and *in vivo* activities than LiHyal (De-Bona et al., 2021). As it was produced by eukaryotic cells, LiHyal2 is post-translationally modified by the addition of high-mannose N-linked carbohydrates.

In fact, other venom hyaluronidases, such as those from *Apis mellifera* (honeybee) and *Brachypelma vagans* (tarantula), were efficiently expressed in baculovirus systems in insect cells (Soldatova et al., 2007; Clement et al., 2012). This expression system has proved to be quite efficient in producing post-translationally modified and active venom hyaluronidases and may be an interesting alternative to purified or recombinant mammal hyaluronidases. In addition, as venom hyaluronidases belong to the same group of enzymes as mammal hyaluronidases, which have already been used as therapeutic agents, they emerge as an alternative source of such remarkable molecules.

### Knottins (Inhibitory Cystine Knot Peptides): Potential Peptides to be Used in the Development of Bioinsectices, Analgesics and Tumor Imaging Agents

Knottins, also known as ICK peptides (Inhibitory Cystine Knot), constitute a family of peptides that characteristically contain cysteine residues forming intramolecular disulfide bonds. These disulfide bonds are arranged to set a pseudo-knot structure, in which a ring established by two disulfide bonds connected to the peptide backbone is intersected by a third disulfide bond (Norton and Pallaghy, 1998; Saez et al., 2010). Knottins have already been identified as components of *Loxosceles* spiders’ venoms through biochemical and molecular biology studies (De Castro et al., 2004; Gremski et al., 2010; Matsubara et al., 2013, 2017; Meissner et al., 2016). Their biological function as venom toxins has been proven to be insecticide, by displaying toxic effects in insects for feeding purpose. Two experimental studies regarding knottins from *L. intermedia* have demonstrated this insecticide activity. First, De Castro et al. (2004), through chromatography approaches, purified a fraction containing three native knottins (identified as LiTx1, LiTx2 and LiTx3) that induced lethal flaccid paralysis on larvae of *Spodoptera frugiperda* (fall armyworm). Additionally, Matsubara et al. (2017) produced a recombinant knottin (U_2_-SCRTX-Li1b) that exhibited long-lasting paralysis in sheep blowflies (*Lucilia cuprina*), which was irreversible even at 72 h post-injection. These exploratory findings unveil the high potential of knottins to be used in the development of bioinsecticides, which constitute an environmentally healthier approach to control pests of economic interest when compared to the chemical pesticides widely used (Windley et al., 2012; King, 2019). Studies regarding knottins from spiders’ venoms have shown that these peptides can be highly selective, being even taxa-specific in some cases (Herzig et al., 2016; King, 2019). This feature makes knottins desirable tools to manage crop pests, since these molecules do not represent harm to insects or other non-target organisms beneficial to the ecosystem (Nakasu et al., 2014). Moreover, it has been described that knottins are, in general, orally active, which means that these toxins are ingested by the insects and are absorbed in the midgut. Some knottins have been engineered in order to be produced as recombinant fusion proteins to molecules (such as lectins) that enhance the absorption by insect’s midgut epithelium (Fitches et al., 2004, 2012; Bonning and Chougule, 2014). Then, these peptides get into the hemolymph and reach their targets, which are ion channels and receptors present in neuromuscular junctions or neurons located in the central nervous system (King, 2019). Due to their “knot” structure, knottins are highly protease-resistant and have been proven to greatly maintain their stability in samples of insect hemolymph (Herzig and King, 2015). In addition to all the already mentioned advantages, spider knottins exert their neurotoxic effects on insects in a fast-manner and are not predicted to bioaccumulate or generate products toxic to the environment after being degraded (King, 2019).

Experimental evidence regarding other applications of knottins from *Loxosceles* spiders remains lacking. However, it is relevant to highlight that *L. intermedia* knottins, for example, have proven to be the most abundant in terms both of transcripts encoded in the venom glands and protein content (together with the phospholipases D) in venom samples analyzed using SDS-PAGE (Gremski et al., 2010; Matsubara et al., 2017). Also, the diversity of these peptides has shown to be even greater as demonstrated by the existence of different knottins encoded in the venom glands of three different *Loxosceles* species (Matsubara et al., 2017). This great representativeness is a strong indication of the existence of multiple targets and, consequently, of multiple applications for these toxins. Thus, another search for the use of knottins from *Loxosceles* is related to their possible analgesic properties, which are suggested by the painless characteristic of the bites in humans (Da Silva et al., 2004; Gremski et al., 2014). Studies regarding knottins from several spiders’ species have shown that these toxins can modulate specific ion channels and receptors associated to the pathophysiological event of pain, inducing analgesic effects. For example, it was shown that two synthetic forms of the knottin peptide μ-TRTX-Df1a (Df1a) from a tarantula (*D. fasciatus*) interacts with a specific region of a voltage-gated sodium channel (Na_v_1.7), which is known to be activated during the pain event in humans and induces analgesia in mice previously treated with a scorpion molecule (OD1) that potentiates the activity of Na_V_1.7 channels (Cardoso et al., 2017; Dongol et al., 2019).

Knottins from spider venoms have also been associated to antifungal (Ayroza et al., 2012), antiarrhythmic (Bode et al., 2001) and antimalarial (Choi et al., 2004) properties. Other spiders’ knottins have been engineered to be used as imaging agents, by producing molecules containing peptide fragments that bind to tumor cells and fluorescent probes; the knottins have been thought to be used in this approach because they are extremely biological- and thermostable in body fluids, exhibit high affinity to their target in minute concentrations, as well as display rapid clearance from non-target tissues/organs, which means that they are efficiently eliminated by the kidneys (Moore et al., 2013; Kintzing and Cochran, 2016). As described, given the diversity of knottins found in the venoms of *Loxosceles* spiders and their respective targets, multiple activities such as those herein mentioned can be investigated in order to determine new uses for these toxins.

### Translationally Controlled Tumor Protein: Target Candidates in Histamine-Related Pathologies and as Pro-Proliferative Biomaterial

A protein from the translationally controlled tumor protein (TCTP) family was described in Loxosceles venoms (Sade et al., 2012). The TCTP protein, also known as histamine-releasing factor (HRF) or fortilin, is a highly conserved and ubiquitous protein, described as multifunctional due to its wide range of function repertoire regarding both intracellular and extracellular biological processes (Bommer and Telerman, 2020). *Loxosceles* TCTP was initially described in *L. intermedia* transcriptome (Gremski et al., 2010). TCTP family proteins have already been described in other arthropods such as in the gland secretion of ixodid ticks (Mulenga and Azad, 2005), in the venom gland of tarantula *Grammostola rosea* (Kimura et al., 2012), and in the venom gland transcriptomic and proteomic analyses of Scytodes spiders (Zobel-Thropp et al., 2014).

The recombinant *L. intermedia* TCTP (LiRecTCTP) produced in a prokaryotic heterologous system causes edema and increases vascular permeability *in vivo* and in animal models (Sade et al., 2012). This protein was related to the inflammatory activity of the venom of *L. intermedia.* Later, TCTP was also found in *L. laeta* transcriptome study, and its presence in *L. gaucho* venom was inferred by immunological cross-reaction studies (Buch et al., 2015). TCTP protein was immunodetected in the whole venom of *Loxosceles* species (*L. intermedia, L. gaucho*, and *L. laeta*) as well as described in the proteomic study of *L. intermedia* venom (Trevisan-Silva et al., 2017). Recently, we have shown that LiRecTCTP acts as a synergistic factor for the PLD actions (LiRecDT1), highlighting its contribution to the pathophysiology of Loxoscelism (Boia-ferreira et al., 2019).

*Loxosceles* TCTP contribution to the exacerbated inflammatory response observed in envenomated patients is related to its histaminergic properties, which suggests that the inhibition of LiTCTP mast cell activation effects could be a therapeutic approach to reduce the inflammatory events responsible for the main symptoms in cutaneous loxoscelism. TCTP was already reported in the biological fluid of asthmatic and parasitized patients (MacDonald, 2012) and human TCTP (54.9% of similarity with LiRecTCTP, using EMBOSS Needle tool) was described as a target for asthma and allergy clinical treatments (Kawakami et al., 2019).

Regarding the multifunctional role of TCTP, LiTCTP is a promising toxin and potential target model in the several cellular processes in which TCTP protein participates (Amson et al., 2013). As TCTP family is highly conserved, we can suggest to LiTCTP some biotechnological applications that have already been described for other TCTP proteins, in the different fields of general biology (toxinology, allergy, parasitology, and oncology) and biomaterial research (dental restoration and drug delivery) as it is discussed hereafter.

In parasitology, TCTP is suggested to be involved in the establishment, maintenance, and pathogenesis of parasite infections. When the *Plasmodium* TCTP (42.2% of similarity with LiRecTCTP) was evaluated as a malaria vaccine, a significant reduction of parasitemia in the early stages of the infection was seen (Taylor et al., 2015). *Plasmodium* TCTP has been shown to bind directly the anti-malarial drug artemisinin (Fujita et al., 2008) and to have higher expression levels on increased drug resistance conditions (Bhisutthibhan et al., 1998).

TCTP proteins harbor, in their N-terminal, a transduction domain (PTD) which could be applied as vehicles in drug delivery systems. These domains are recognized as promising vehicles for the delivery of macromolecular drugs. Different studies showed the effectiveness of the TCTP carrier peptide (residues 1-10, MIIYRDLISH) internalization in different cell types (Bae and Lee, 2013; Maeng et al., 2013; Calderón-Pérez et al., 2014). A modified version of TCTP PTD was shown as a promising vehicle for intranasal delivery of insulin (Bae et al., 2018). More recently, TCTP was shown to translocate into oocytes across the zona pellucida (ZP) and to prevent quality deterioration during *in vitro* culture. This data is quite important as the delivery of exogenous molecules into mammalian oocytes or embryos has been a challenge because of the existence of the protective ZP surrounding the oocyte membrane (Jeon et al., 2017).

TCTP proteins present proliferative properties and anti-apoptotic activity, and TCTP from *Fenneropenaeus merguiensis* (banana prawn) was explored as supplements in dental restorative materials (Wanachottrakul et al., 2011). This TCTP was shown to promote osteoblast cells proliferation, differentiation and function, highlighting its potential use as a supplementary medical material in dentistry (Sangsuwan et al., 2015; Kedjarune-Leggat et al., 2020). TCTP ability to protect cells in a range of stress conditions was recently described in cardiomyocytes (Cai et al., 2019). Results have shown that TCTP plays a critical role for the survival of these cells and has a protective function against drug-induced cardiac dysfunction in mice.

TCTP’s collection of molecular partners and involvement in different biological events is behind this protein’s biotechnological potential, which exploration has just initiated.

### Phospholipases D: Candidates in the Development of Antibacterial Drugs and Tools to Comprehend the Regulation of Some Biological Processes

Phospholipases D are enzymes that have a broad range of biological activities and are involved in the regulation of many pathological processes including those related to tumor cells (Houben and Moolenaar, 2011). The protein Autotaxin (ATX), for example, is a phospholipase D with activity on lysophosphatidylcholine and it is responsible for the formation of lysophosphatidic acid (LPA) in the blood and other tissues. ATX is highly expressed in several tumors, and the lipid mediator released (LPA) evokes responses such as cell migration and proliferation, as well as and survival for a wide range of tumor cells (Tania et al., 2010; Houben and Moolenaar, 2011). The *L. intermedia* PLD, LiRecDT1 can trigger some biological responses on melanoma cells, such as proliferation and calcium influx, besides binding to the membrane of B16F10 cells (Wille et al., 2013). A study that investigated the activity of the *L. gaucho* PLD LgRec1 on B16F10 cells showed that this toxin reduces the viability of those cells, thus presenting a potential antitumor activity (Siqueira et al., 2019). The authors potentialized this activity by fusing this LgRec1 with a snake venom disintegrin called Echistatin. The resulting hybrid protein, named Rechistatin, was more efficient to promote cell death than only Echistatin or only LgRec1 since the disintegrin portion helped to deliver the PLD to the target cell (Siqueira et al., 2019). A deeper investigation regarding this activity of *Loxosceles* PLDs still remains to be done. In addition, as ATX, LiRecDT1 can be a potential exogenous tool to better understand the regulation of biological processes in tumor cells and in cell membranes.

Besides the potential use of *Loxosceles* PLDs to study tumor cells and their activities, other fields to apply these toxins as useful components have been explored. Currently, the resistance of some bacteria to antibiotics is a major problem to the health care systems. Thus, the development of new drugs with antibacterial activity has become an emerging area (Echols, 2012; Sudarshan and Dhananjaya, 2016). Peptides present in venoms of animals such as scorpions, snakes and spiders are potential sources of such molecules (Siqueira et al., 2019). In this sense, *Loxosceles* venoms’ PLDs may also serve as potential antimicrobial drugs. A peptide from the venom of *L. gaucho* (U1-SCRTX-Lg1a) showed antibacterial activity in gram-negative bacteria, such as *Pseudomonas aeruginosa* and *Enterobacter cloacae*, and did not affect the viability of HeLa cells. There is evidence that these peptides arise from a limited proteolytic cleavage of PLDs, since it shows great similarity with some regions of the amino acid sequences of nine *Loxosceles* PLDs (Segura-Ramírez and Silva Júnior, 2018).

## Perspectives

Brown spider venom presents a range of natural proteins and peptides that could eventually find their way into pharmaceuticals, biological agents and tools for the development of novel therapies. The potential applications of such molecules and the search for novel therapies to treat loxoscelism will continue to stimulate the studies regarding this complex venom, which will certainly result in novel approaches to overcome various challenges in a foreseeable future.
